# Production of persimmon and mandarin peel pastes and their uses in food

**DOI:** 10.1002/fsn3.2146

**Published:** 2021-01-23

**Authors:** Yoko Tsurunaga, Taido Takahashi, Yoshiaki Nagata

**Affiliations:** ^1^ Shimane University Matsue Japan; ^2^ The University of Shimane Matsue Japan; ^3^ Shimane Institute for Industrial Technology Matsue Japan

**Keywords:** folin assay, fruit peel pastes, mandarin, persimmon

## Abstract

Fruit peels are often produced as a byproduct of processing and are usually disposed of as industrial waste. We conducted a study on the effective use of peels for the food industry using persimmons and mandarins as models. The production of persimmon and mandarin peel pastes, their flavor components, color, polyphenol contents, physical properties, and uses in foods (jam, cookies, and madeleines) were studied. The effects of heat treatment for sterilization, to effectively use persimmon and mandarin peels, were also investigated. The amount of water added to produce the optimum persimmon and mandarin peel pastes was 0.5× and 2.0× the weight of the respective peel samples. The main flavor components, as per GC‐MS spectra of persimmon and mandarin peel pastes, were 4 and 1, respectively. The Folin assay showed the polyphenol contents of persimmon and mandarin pastes as 33.9 mg and 236.3 mg of catechin equivalent per 100 g of fresh fruit, respectively. The persimmon paste specifically improved the physical properties of cookies, whereas the mandarin paste was well suited to all the processed food forms. Heat treatment for sterilization decreased cohesiveness but increased breaking strength and adhesiveness in persimmon and mandarin pastes.

## Graphical abstract

Pastes were prepared using Persimmon and mandarin fruit peels, and their heat sterilization property and usefulness as an additive to processed foods were evaluated.

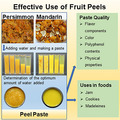

## 1 INTRODUCTION

Recently, a shift has occurred in food production, where items once considered wastes are increasingly being used in various forms. For example, seeds, peels, and immature fruits are being studied extensively as unused resources in the food industry. Fruit peels are rich in functional components such as polyphenols, carotenes, and vitamins. In this study, we examined the uses of persimmon (*Diospyros kaki* Thunb.) and mandarin (*Citrus unshiu* Marcow.) peels, which are typically treated as wastes.

In Japan, it is customary to process astringent persimmons to a dried form. During the production of dried persimmons, a large amount of peels results as a byproduct. Persimmon peels have exhibited antioxidant activity in cholesterol‐fed rats (Gorinstein et al., 1998), and its polyphenols have protective effects against glucose‐induced oxidative stress (Yokozawa et al., 2007). Mandarins are used to make juices and canned foods; however, the peels are disposed of during the manufacturing processes. Cho (2003) reported that the mandarin peel contains a large amount of pectin. Successful concentration and purification of pectin from mandarins using a crossflow microfiltration system have been demonstrated (Cho, 2003). Furthermore, mandarin peels contain several polyphenols, such as hesperidin, nobiletin, and tangeretin, which have anti‐neuroinflammatory effects (Ho & Kuo, 2014).

Here, we aimed to effectively use persimmon and mandarin peels, which are treated as industrial wastes despite their many useful components. First, we determined the optimum conditions to produce persimmon and mandarin peel pastes, including the amount of added water required. Next, we examined the effects of heat treatment on the color, soluble polyphenol contents, and physical properties of the peel pastes. Finally, we manufactured jam, cookies, and madeleines using the persimmon and mandarin peel pastes, and performed sensory evaluation tests to determine their influence on the physical properties, taste, and aroma of the foods. The results indicated that the quality of persimmon and mandarin peel pastes is not considerably affected by heat treatment and that the pastes may be used as suitable additives to processed foods.

## 2 MATERIALS AND METHODS

### 2.1 Materials

**FIGURE 1:**
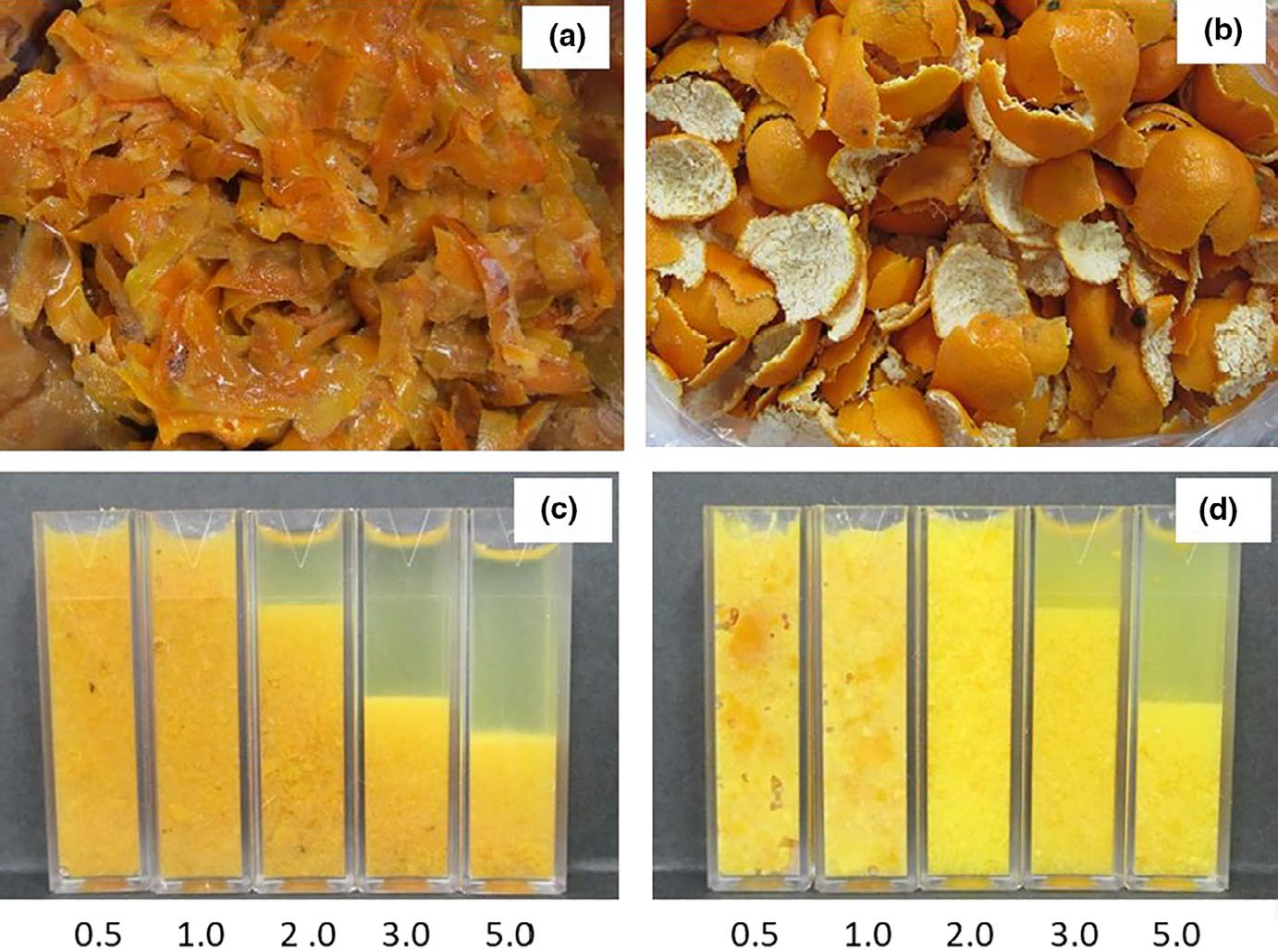
Starting materials for (a) persimmon and (b) mandarin peel pastes, and water separation after addition of 0.5, 1.0, 2.0, 3.0, and 5.0 × water to (c) persimmon and (d) mandarin peel pastes

Persimmon fruits were harvested from persimmon “Saijo” trees (*D. kaki* Thunb.) in Shimane prefecture in Japan in November 2015. Mandarin fruits were harvested from mandarin “Unshu” trees (*C. unshiu* Marcow.) in Wakayama prefecture in Japan in December 2015. The peels of both fruits were used in the experiments (Figure 1). Both persimmon and mandarin fruits used in the experiment were fully ripe. The persimmon peel, which is usually discarded, produced during the production of dried persimmons, was used. Therefore, the astringent removal process commonly conducted with persimmons is not implemented.

### 2.2 Optimization of paste production

Due to the water content in the peels of persimmon and mandarin being very low, it is difficult to produce pastes from the peels without adding water. Therefore, we determined the optimal amount of water required for processing. Persimmon and mandarin peels were first roughly chopped using a food processor (PS‐3000S, Chubu.co, Nagoya, Japan). The peels were then combined with 0.5, 1, 2, 3, or 5× the water (w/w) and ground to a smooth paste using a 1 L Osterizer 16‐speed blender (Sunbeam Oster). For every sample group, 200 g of peel paste was used.

### 2.3 Analysis of flavor components

Analysis of the flavor components was performed by modifying methods of Farmeti (Farneti et al., 2012). To analyze flavor components, both peel pastes were enclosed in a 20 ml head‐space vial and warmed for 5 min at 60°C. Then, a solid‐phase microextraction (SPME) fiber was inserted in the vial and the components were extracted for 30 min at 60°C. The SPME fiber was inserted into an ISQ™ QD Single Quadrupole Gas Chromatography‐Mass Spectrometry (GC‐MS) System incorporating a TRACE™ 1,310 Gas Chromatograph and a TriPlus RSH™ Autosampler (all from Thermo Fisher Scientific), and GC‐MS analysis was performed using the conditions listed in Table 1. Compounds in the obtained particle spectra were estimated using the National Institute of Standards and Technology (NIST) 11 Library for Mass Spectrometry and Odor Search (Alpha MOS).

**TABLE 1:** Gas chromatography‐mass spectrometry (GC‐MS) analytical conditions

GC‐MS equipment	ThermoFisher Scientific Trace1310GC, ISQ QD, TriPlus RSH	
Solid‐phase microextraction condition	Fiber	supelco DVB/CAR/PDMS 10 mm
	Extracting method	60°C for 30 min
GC condition	Carrier gas	Helium, constant pressure 150 kPa
	Column	TG‐WAXMS 0.25 mm × 60 m, 0.25 μm
	Vaporizing chamber	splitless 1 min 250°C
	Temperature program	50°C for 4 min—temperature rising at 5°C/min—250°C for 15 min
MS condition	Scanning range	30–400 (m/z)
	Ion source	200°C
	Transfer line	250°C

### 2.4 Color assessment

Color was measured in three categories: L∗, black to white; a∗, red to green; and b∗, yellow to blue. L*, a*, and b* values for the peel pastes were measured using a spectrum colorimeter (CR‐13, Konica Minolta). The results are expressed as the mean ± standard error (*SE*; *n* = 6).

### 2.5 Soluble polyphenol contents

To analyze the soluble polyphenol contents, 20 ml of 80% methanol was added to 5 g of the peel paste sample, and the mixture was homogenized to obtain the extract. The 80% methanol extract was filtered to obtain a sample solution. Each sample solution was diluted 10–50 times, and the soluble polyphenol content was measured using the Folin method (Goldstein & Swain, 1965) and analyzed by partially modifying the method proposed by Chung & Seong (Chung et al., 2015). The results were expressed proportionally in terms of mg (+) catechin per 100 g of fruit fresh weight (mg/100 g FW).

### 2.6 Physical properties

Physical properties of the samples were measured using an RE2‐33005B Creep Meter (YAMADEN Co., Ltd.) in a stainless steel dish. Breaking strength, breaking energy, and breaking strain were measured using a 2‐cm‐diameter cylindrical plunger, with a velocity of 5 mm/s and gauge factor of 66.67%. Six peel paste samples were assessed in each sample group. Measurements were obtained using a 20 N load cell at 20 ± 1°C (Takei et al., 2016).

### 2.7 Production of food products containing fruit peel pastes

Jams, cookies, and madeleines were prepared using the persimmon and mandarin peel pastes. Jam, cookies, and madeleines were all made using common quantities and methods. A persimmon paste (peel and fruit), with the astringency removed using dry ice for 4 days, was used as a control. Whole mandarin (peel and fruit) paste was used as a control for the mandarin peel paste. Both control pastes contained no added water. The persimmon and mandarin peel paste samples contained 0.5× and 2.0× water, respectively. To prepare the jam, 75 g of the fruit paste, 20 g sugar, and 50 ml water were cooked in a pot over medium heat for approximately 15 min. The heat was then turned off, and 30 ml of lemon juice was mixed in. The jam was poured into jars and refrigerated at 4°C in airtight containers for storage. The cookies were made using 200 g wheat flour, 25 g liquid egg, 120 g unsalted butter, 60 g granulated sugar, 2 g salt, and 75 g of the fruit peel paste. The butter and salt were mixed for 30 s in a mixer (DB‐2263, Kai Co., Ltd., Tokyo, Japan), and then, granulated sugar was added and mixed for 5 min. After adding the liquid egg and peel paste and mixing for 2 min, the flour was added in, and the mixture was combined for an additional 2 min. The cookie dough was wrapped in cling film and chilled at 4°C for 30 min, then rolled into 2 cm‐diameter logs, wrapped in cling film, and frozen at −25°C for 2 hr. The dough was then cut into 6 mm thick cookies immediately before baking at 170°C for 20 min. The madeleines contained 100 g wheat flour, 100 g liquid egg, 60 g unsalted butter, 90 g sugar, and 75 g fruit peel paste. The liquid egg, fruit peel paste, sugar, and butter were mixed using a mixer (DB‐2263, Kai Co., Ltd.) for 7 min. The flour was then added, and the mixture was combined for an additional 2 min. The madeleine batter was poured into aluminum foil molds and baked for approximately 20 min at 180°C.

### 2.8 Sensory evaluation

Ten students from Shimane University were enlisted to perform sensory evaluations. All volunteers were experienced in the food evaluation procedure. Appearance, taste, physical properties, and total evaluation were scored using a 7‐point scale, using 3 points for good, 0 points for average, and −3 points for bad.

### 2.9 Heat treatment for sterilization

We examined the influence of heat treatment on the color, soluble polyphenol contents, and physical properties of each peel paste, as heat sterilization is necessary to circulate the fruit peel paste. The persimmon and mandarin peel pastes contained 0.5× and 2.0× added water, respectively. The heating temperature and time were at 80 and 90°C, for 30 and 60 min, respectively, with some modifications of Sandoval et al. methods (Sandoval et al., 1994). First, 100 g of peel paste was enclosed in a bag, and heat treatment was performed in a water bath, maintained at a constant temperature (BS400, Yamato Scientific Co., Ltd.). Following heat treatment, samples were immediately cooled on ice and evaluated.

## 3 RESULTS AND DISCUSSION

### 3.1 Production of the peel pastes

The persimmon and mandarin peel pastes required different quantities of water to be added to them for suitable pastes to be developed (Figure 1). Based on the appearance and liquid release, 0.5× and 2.0× water was optimal for the persimmon and mandarin peel pastes, respectively. To produce the persimmon peel paste, 100 g of water was added to 200 g of the peel. To produce the mandarin peel paste, 400 g of water was added to 200 g of peel. The rind of the mandarin also contains a part called albedo (Ballester et al., 2013; Nadi et al., 2019), which has a low water content. Therefore, it is inferred that more water was needed to make the paste than for persimmons.

### 3.2 Color, polyphenol contents, and physical properties of the peel pastes

The color, polyphenol contents, and physical properties of the persimmon and mandarin peel pastes were examined. When the colors of the two peel pastes were compared, the a* value of the peel paste of persimmon (11.5 ± 0.1) was seen to be higher than mandarin (7.1 ± 0.1). The L* and b* values of the mandarin peel paste were higher (54.0 ± 0.1 and 42.2 ± 0.5, respectively) than persimmon (35.6 ± 0.1 and 26.0 ± 0.3, respectively). The persimmon peel paste scored high for red coloring, and mandarin peel paste scored high for yellow. The Saijo persimmon used in this study is the most popular persimmon in Japan (Toyota et al., 2016). Persimmons are also known to change color from orange to red‐orange around November (Hu et al., 2013). The Wenzhou mandarin is the main fruit in Japan (Hisanaga et al., 2018; Inoue et al., 2010) and ripens, turning yellowish, around December (Kitajima et al., 2007). As seen in Figure 1, the inner part of the peel of citrus fruits, such as the Wenshu mandarin, has a white part called albedo (Ballester et al., 2013; Nadi et al., 2019). The albedo was mixed in when the fruit is pasted, giving the bright color. Astringent persimmon fruits are rich in soluble polyphenols; however, in this experiment, their peels contained minimal soluble polyphenols (33.9 ± 1.9); regardless, we did not perform de‐astringency processing. The soluble polyphenols of persimmon are primarily tannins, which exist in vacuoles (Salvador et al., 2007). In contrast, mandarin peels contain high levels of soluble polyphenols, 236 mg catechin/100 g FW. Mandarin peels contain several functional compounds, such as nobiletin (Gaydou et al., 1987), which has curing effects in dementia (Yamakuni et al., 2010) and allergy (Onishi et al., 2014). This makes mandarin peels significant in the industrial, pharmaceutical, and food fields. Regarding their physical properties, the mandarin peel paste displayed higher values for hardness, cohesiveness, and adhesion (853.1 ± 22.1, 0.89 ± 0.01, and 214.9 ± 9.4, respectively) than the persimmon peel paste (655.7 ± 13.8, 0.72 ± 0.01, and 109.9 ± 3.0, respectively).

### 3.3 Volatile components of persimmon and mandarin peel pastes

**FIGURE 2:**
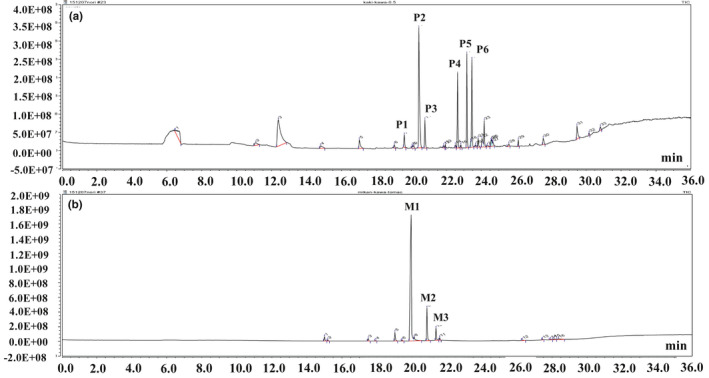
Gas chromatography‐mass spectrometry chromatograms of (a) persimmon and (b) mandarin peel pastes

GC‐MS spectra for the persimmon and mandarin peel pastes have been shown in Figure 2(a,b), respectively. Aromatic compounds in each peak were identified using Odor Search, a database of smell components. The peak at 19.57 min (P1) retention time (RT) in the persimmon peel paste was identified as D‐limonene, the major volatile component in the fruit. The RT peak at 20.43 min (P2) was 2‐hexenal, which smells like sweet almond; the RT peak at 20.77 min (P3) was tetrahydroionyl acetate, which smells like berries; the RT peak at 22.64 min (P4) was 1‐hexanol, which smells fruity and alcoholic; the RT peak at 23.17 min (P5) was 3‐hexen‐1‐ol, which smells like cut grass; and the RT peak at 23.46 min (P6) was 2‐hexen‐1‐ol, which smells fruity and leafy. In the mandarin peel paste, the RT peak at 19.57 min (M1) also corresponded to D‐limonene. The RT peak at 20.86 min (M2) was terpinene, which smells woody and resinous, and the RT peak at 21.37 min (M3) was terpinolene, which smells of pine and citrus fruit. Generally, food contains several volatile components; however, the marked peaks in the persimmon and mandarin peel pastes were 4 and 1, respectively, indicated by the characteristic spectra.

### 3.4 Production of foods containing the peel pastes

We prepared jam, cookies, and madeleines using the persimmon and mandarin peel pastes. The results of the sensory evaluation are shown in Table 2. Sensory evaluation of the jam produced using the persimmon peel paste was unfavorable in all aspects of the evaluation, and the paste was hence deemed unsuitable for jam production. Conversely, the mandarin peel paste jam obtained favorable evaluation values, even higher than those obtained with the whole mandarin fruit paste. Cookies made with the persimmon peel paste demonstrated better evaluation than the persimmon fruit control in all the values except for appearance, and the addition of the persimmon peel paste produced a good cookie. The mandarin peel paste cookie demonstrated a favorable evaluation compared to the control. These results emphasize the utility of adding persimmon and mandarin peel pastes to cookies. Madeleines made with the mandarin paste demonstrated better evaluation than those made with the persimmon paste for all criteria evaluated. Altogether, the results demonstrate that the suitability of the pastes depends on the type of processed food. The persimmon peel paste specifically improved the physical properties of cookies, whereas the mandarin peel paste was well suited to all the processed food forms in this study.

**TABLE 2:** Sensory evaluation of persimmon and mandarin peel pastes (*n* = 10)

Kind of Paste	Jam				
	Appearance	Taste	Smell	Texture	Total
Control of persimmon	0.6 ± 0.6	0.1 ± 0.6	0.3 ± 0.5	0.0 ± 0.6	0.9 ± 0.5
Peel of persimmon	−2.0 ± 0.3	−1.0 ± 0.5	−0.1 ± 0.5	−1.0 ± 0.5	−1.1 ± 0.4
Control of mandarin	1.8 ± 0.2	1.8 ± 0.2	1.3 ± 0.5	0.6 ± 0.5	1.8 ± 0.3
Peel of mandarin	2.3 ± 0.2	1.1 ± 0.5	1.4 ± 0.5	0.6 ± 0.6	2.0 ± 0.3

Kind of Paste	Cookie				
	Appearance	Taste	Smell	Texture	Total
Control of persimmon	1.0 ± 0.5	0.3 ± 0.7	1.0 ± 0.4	0.6 ± 0.5	0.6 ± 0.7
Peel of persimmon	0.9 ± 0.4	1.5 ± 0.4	1.3 ± 0.3	1.6 ± 0.3	1.9 ± 0.2
Control of mandarin	0.5 ± 0.5	1.6 ± 0.5	2.0 ± 0.4	1.1 ± 0.2	1.5 ± 0.3
Peel of mandarin	1.1 ± 0.5	2.0 ± 0.3	2.4 ± 0.3	1.3 ± 0.5	2.5 ± 0.2

Kind of Paste	Madeleine				
	Appearance	Taste	Smell	Texture	Total
Control of persimmon	0.6 ± 0.4	0.4 ± 0.4	0.5 ± 0.41	−0.8 ± 0.6	0.0 ± 0.5
Peel of persimmon	−0.1 ± 0.5	0.4 ± 0.5	0.6 ± 0.4	−0.5 ± 0.7	0.8 ± 0.8
Control of mandarin	1.6 ± 0.4	1.4 ± 1.8	1.6 ± 0.2	0.8 ± 0.4	1.8 ± 0.3
Peel of mandarin	2.0 ± 0.2	1.8 ± 0.4	1.5 ± 0.3	1.1 ± 0.4	2.0 ± 0.3

Note

Control for persimmon: Whole fruit + peel with astringent removal.

Control of mandarin: Whole fruit + peel.

Appearance, taste, physical properties, and total evaluation were scored on a 7‐point scale, using 3 points for good, 0 points for average, and −3 points for bad.

Values are expressed as mean ± standard error (*n* = 10).

### 3.5 Peel paste thermal stability

**FIGURE 3:**
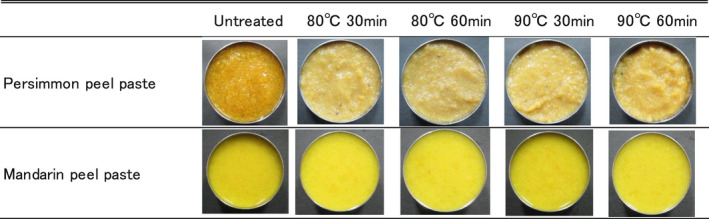
Effect of heat treatment on the appearance of persimmon and mandarin peel pastes

**FIGURE 4:**
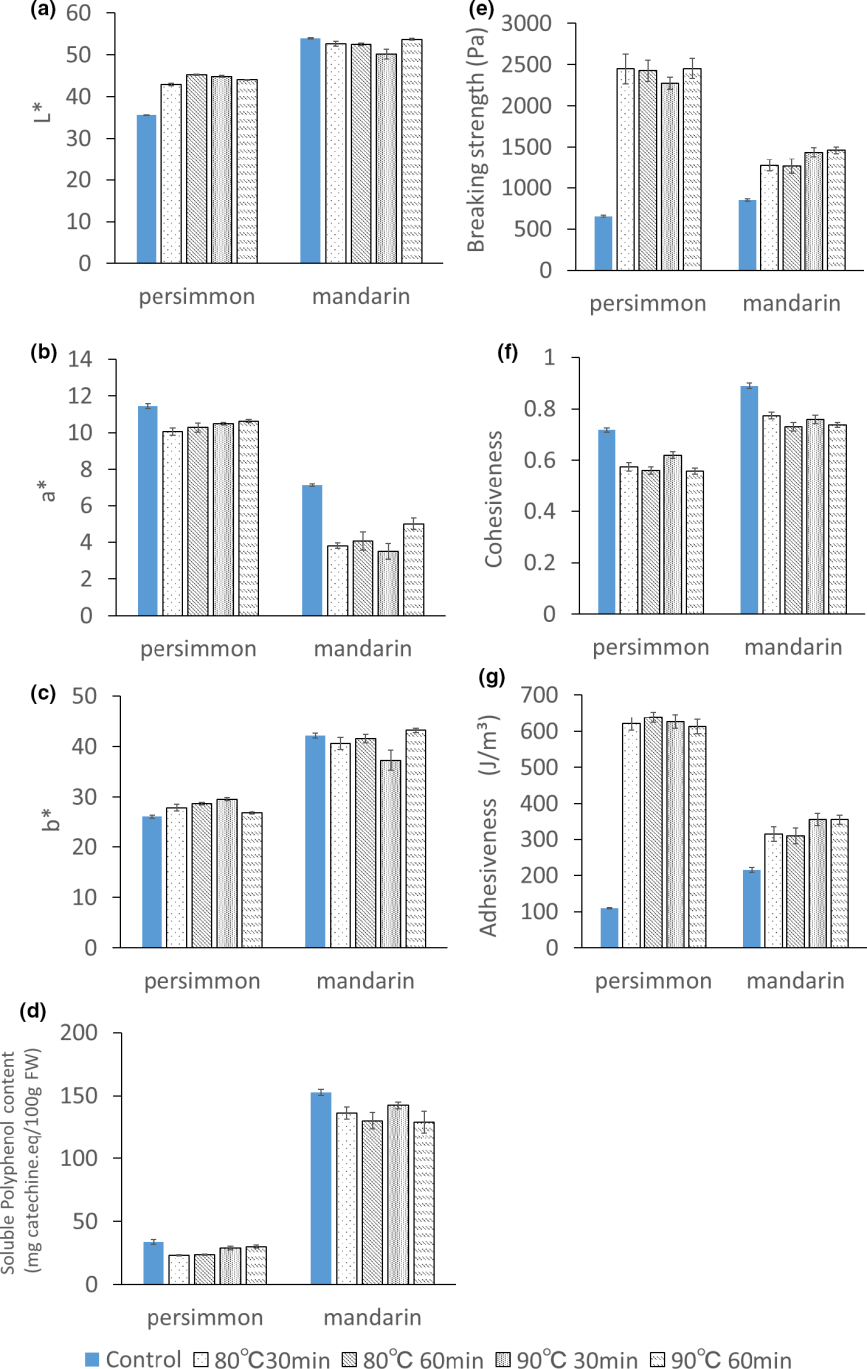
Effect of heat treatment on the color (a, b, c), the soluble polyphenol content, (d) and the physical properties (e, f, g) of persimmon and mandarin peel pastes. Bars represent the mean ± standard error. In (a)–(d) *n* = 6, and in (e)–(g) *n* = 10

After heat sterilization at 80 or 90°C, the persimmon peel paste acquired a darker color tone and became clumpy, whereas the mandarin peel paste appeared unaffected (Figure 3); however, fading of the redness was confirmed by a decrease in the a* value (Figure 4b). The L* value, which indicated that the brightness of the persimmon peel paste, increased after heat treatment, whereas the influence of heat treatment on the L* and b* values of the mandarin peel paste was minimal (Figure 4b,c). The persimmon fruit is usually treated with carbon dioxide or alcohol to remove free tannins, decreasing its astringency. Heat treatment during food processing normally increases the astringency of fruits. However, we did not observe an increase in astringency after heat treatment of the persimmon fruit paste, reducing the need for further processing (Figure 4d). Heat treatment of the mandarin peel paste had a minimal effect on the soluble polyphenols. Both persimmon and mandarin peels contain β‐cryptoxanthin, a type of carotene. Fading of the red color in both pastes suggests the reduction of cryptoxanthin in persimmon and mandarin peel pastes due to heat treatment.

### 3.6 Effect of heat treatment on the physical properties of the peel pastes

Figure 4 shows the breaking strength (E), cohesiveness (F), and adhesiveness (G) of the persimmon and mandarin peel pastes. The breaking strength of the control and heat‐treated (80 or 90°C, for 30 or 60 min) persimmon peel pastes was 656 Pa, 2,73–2,453 Pa, respectively, and 853 Pa and 1,264–1,461 Pa, respectively, for mandarin. The breaking strength value after heat treatment was high, suggesting that it increased the firmness of both peel pastes. The cohesiveness of the control and heat‐treated persimmon peel pastes was 0.71 and 0.56–0.62, and 0.89 and 0.73–0.76, respectively, for mandarin. The adhesiveness of the control and heat‐treated persimmon peel paste was 110 J/m^3^ and 613 J/m^3^–638 J/m^3^, and 214 J/m^3^ and 309 J/m^3^–355 J/m^3^, respectively, for mandarin. These results indicate that the adhesiveness of both peel pastes increased upon heating, particularly that in persimmon. Soluble pectin is present between the matured fruit and its peel, and is easily eluted by heating, allowing its use in jams and jellies. Therefore, heat sterilization was performed assuming that the pectin would be distributed in the paste. However, the heating may instead have induced the elution of pectin from the peel paste, altering its physical properties.

## 4 CONCLUSIONS

We characterized the optimum conditions to produce fruit peel pastes from persimmons and mandarins. The amount of water required for the production of an optimum paste from fruit peels was 0.5× and 2.0× for the persimmon and mandarin peel, respectively. The color of the persimmon peel paste scored high for red coloring, and the mandarin peel paste scored high for yellow. In addition, the persimmon peel paste specifically improved the physical properties of cookies, whereas the mandarin peel paste was well suited to all the processed food forms. This study demonstrates the production of persimmon and mandarin peel pastes and their uses in the food industry.

## CONFLICT OF INTEREST

The authors declare that they do not have any conflict of interest.

## ETHICAL APPROVAL

This study does not involve any human or animal testing.

## Data Availability

The data that support the findings of this study are available from the corresponding author upon reasonable request.
